# Development of a Sequential Injection Analysis System for the Determination of Saccharin

**DOI:** 10.3390/s17122891

**Published:** 2017-12-12

**Authors:** Budi Wibowotomo, Jong-Bang Eun, Jong Il Rhee

**Affiliations:** 1 Department of Food Science and Technology and Functional Food Research Center, Chonnam National University, YongBong-Ro 77, Gwangju 61186, Korea; budiwee@yahoo.com (B.W.); jbeun@jnu.ac.kr (J.-B.E.); 2 Department of Industrial Technology, The State University of Malang, Jl Semarang 5, Malang 65145, Indonesia; 3 School of Chemical Engineering and Research Center for Biophotonics, Chonnam National University, YongBong-Ro 77, Gwangju 61186, Korea

**Keywords:** p-chloranil, food-product processing, hydrogen peroxide, saccharin, sequential injection analysis

## Abstract

Saccharin is a powerfully sweet nonnutritive sweetener that has been approved for food-processing applications within the range of 100–1200 mg/kg. A simple, rapid, and cost-effective sequential injection analysis (SIA) technique was developed to determine the saccharin level. This method is based on the reaction of saccharin with p-chloranil in an ethanol medium with a hydrogen peroxide (H_2_O_2_) acceleration, and the resultant violet-red compound was detected using a UV-Vis spectrophotometer at λ_max_ = 420 nm. To ascertain the optimal conditions for the SIA system, several parameters were investigated, including buffer flow rate and volume, p-chloranil concentration, and reactant volumes (saccharin, p-chloranil, and H_2_O_2_). The optimum setup of the SIA system was achieved with a buffer flow rate, buffer volume, and draw-up time of 1.2 mL/min, 2900 µL, and ~145 s, respectively. The optimal p-chloranil concentration is 30 mM, and the best reactant volumes, presented in an ordered sequence, are as follows: 30 µL of H_2_O_2_, 450 µL of saccharin, and 150 µL of p-chloranil. The optimized SIA configuration produced a good linear calibration curve with a correlation coefficient (R^2^ = 0.9812) in the concentration range of 20–140 mg/L and with a detection limit of 19.69 mg/L. Analytical applications in different food categories also showed acceptable recovery values in the range of 93.1–111.5%. This simple and rapid SIA system offers great feasibility for the saccharin quality control in food-product processing.

## 1. Introduction

Saccharin (C_7_H_5_NO_3_S) is a powerfully sweet nonnutritive sweetener; it is a white crystalline powder with an intensely sweet taste [1] that is 300–500 times sweeter than sugar. Saccharin is characterized by its high solubility, stability, and bitter metallic aftertaste [2]. The excellent stability of saccharin during food processing makes it ideal for many different products. It is applied in a wide range of food products including baked goods, beverages, soft drinks, sugar preserves and confectioneries, vinegar, pickles and sauces, dairy products, fruit, vegetables, nut products, sugar-free jams and marmalades, and low-calorie preserves [3]. It is particularly useful in cases where heat processing is required (e.g., jams, canned products, etc.).

After saccharin was ruled out as a potential carcinogen, the extent of its application in food processing has increased. Prior to 1977, before the publication of the additional studies that failed to support the initial assessments of the carcinogenic effects of saccharin, the United States Food and Drug Administration (US FDA) approved a prohibitionary action regarding the use of sodium saccharin (C_7_H_5_NO_3_S) in dietary products and drugs, which was later repealed [4]. Further, the European Union (EU) via the European Scientific Committee for Food thoroughly assessed the safety of saccharin, considering its relevance in economic and social terms and the limitation of its content in foodstuffs, before it authorized the addition of saccharin to food products and beverages. Presently, however, sodium saccharin is approved in more than 90 countries globally and is widely used in many pharmaceutical and dietary products despite an ongoing controversy regarding its safety [5]. The acceptable daily intake (ADI) values that have been declared for saccharin by the United Nations (UN) Joint Food and Agriculture Organization FAO/World Health Organization (WHO) Expert Committee on Food Additives (JECFA) are within the body-mass range of 0–5 mg/kg [6]. The maximum residue levels (MRLs) of saccharin in different foods have been set within the range of 80–2500 mg/kg [7]. Therefore, various analytical techniques have been applied to determine the saccharin concentrations in numerous pharmaceutical and dietary products. High-performance liquid chromatography (HPLC) has been widely used for the determination of saccharin levels [8,9,10], but this technique requires expensive equipment and a complex sample pretreatment.

In recent years, flow injection analysis (FIA) has received much attention as an advanced analytical procedure. The several distinct advantages of this technique are in terms of its simplicity, cost performance, flexibility, and rapidity [11]. Saccharin concentration has been determined using FIA systems with many different analytical techniques. Saccharin has been precipitated as a mercurous saccharinate, and the mercurous cation was then potentiometrically measured using a silver (Ag)-wire coated with a mercury film. An FIA system consisting of an Ag-wire electrode was developed for the measurement of saccharin concentrations within the range of 2–10 mM and at the sampling frequency of 60 1/h [12]. The precipitation reaction of Ag(I) ions with saccharin has been used to develop an FIA with a merging zone for the determination of saccharin concentration within the range of 2.4–9.64 g/L [13]. An atomic-absorption spectrometry was coupled with an FIA system for the determination of the saccharin concentration, where a continuous flow-manifold-based Ag-ion precipitation was used. The FIA method allows for the determination of the saccharin concentration in the range of 5–75 μg/mL and with a rate of ca. 20 samples/h [14]. In addition, an integrated solid-phase spectrophotometer–FIA method was developed based on an on-line aspartame preconcentration and a separation from the saccharin in a sweetener mixture. With a sampling frequency of 10 1/h, the applicable saccharin concentration range is from 1.0–200.0 μg/mL [15].

The sequential injection analysis (SIA) system, however, is advantageous over the FIA method owing to its versatility and reliability along with its low frequency of maintenance [11]. Further, the potential of the SIA technique means that its operability can be fully automated, with a computer-based control for the aspiration or dispensation of samples and reagents. With respect to food applications, greatly advantageous SIA-system characteristics have been offered, including an easy adaptation to different analytical situations without the need for a physical reconfiguration, an automated sample injection, and a controlled dispersion. The SIA system allows for a choice of different types of liquid driver, a reproducible timing, a great capacity in relation to the solution-handling operations, and it is more flexible for its application in stopped-flow and reversed-flow operations [16,17]. To our knowledge, however, an automated determination of the saccharin concentration for which a SIA system is employed has not been reported; therefore, a simple and rapid SIA method for a quantitative determination of the saccharin concentration in food-product samples is proposed in the present paper. This method is based on the reaction of saccharin with p-chloranil in an ethanol medium together with a hydrogen peroxide (H_2_O_2_) acceleration, whereby a violet-red compound is produced. The SIA system offers great feasibility regarding the determination of the saccharin concentration as it is simple, rapid, and economical; furthermore, it can be used for the quality control of nonnutritive sweeteners in food products.

## 2. Materials and Methods

### 2.1. Reagents

All of the chemicals used in this study were of analytical grade. The saccharin stock solution (C_7_H_4_NNaO_3_S·2H_2_O, 10 mM) was prepared by dissolving 0.1206 g of sodium saccharin in 10 mL of absolute ethanol via a 15-min stirring that was followed by diluting to 50 mL with the same solvent. A 20-mM p-chloranil (Sigma Co., St. Louis, MO, USA) solution was prepared daily by dissolving 0.123 g of p-chloranil in 25 mL of acetone (Mallinckrodt Co., St. Louis, MO, USA). A H_2_O_2_ solution (7.10 M) was prepared from a 30% H_2_O_2_ concentrate (Merck Co., Darmstadt‎, Germany) via a convenient dilution in the ethanol (Mallinckrodt Co., St. Louis, MO, USA). A sodium phosphate-buffer solution (200 mM) was used as the carrier solution.

### 2.2. Sample Preparation

An aliquot of 1.0 or 2.0 g or mL of food was transferred to a 50-mL Erlenmeyer flask. Then, 15 mL of ethanol was added to the flask, followed by a stirring of the mixture for 15 min and a filtration using the Whatman No. 41 paper (Sigma-Aldrich Co., Seoul, Korea). After the repetition of this step (15 mL of ethanol, 15 min) and a third extraction (10 mL of ethanol, 5 min) for a total of 40 mL of the solvent, the filtrate was collected and evaporated until 10 mL remained, and this remaining amount was then cooled in an ice bath for 5 min. Finally, the resultant solution was filtered, transferred to a volumetric flask of 25 mL, and then diluted to the 25-mL volumetric indicator mark using the same solvent.

### 2.3. The SIA System

**Figure 1:**
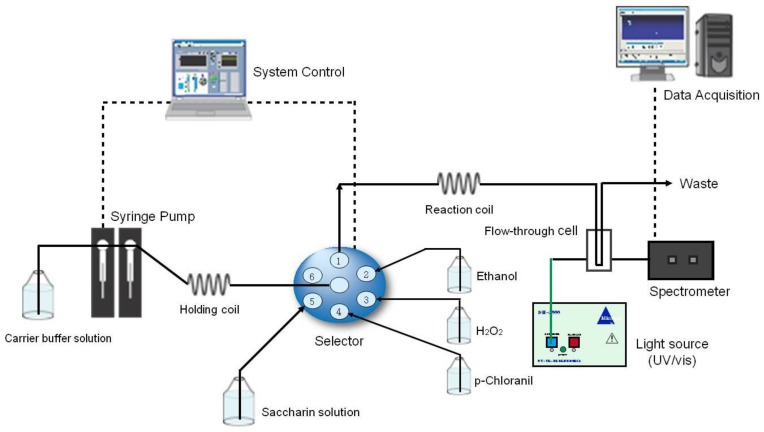
Schematic diagram of an sequential injection analysis (SIA) system for the determination of the saccharin concentration.

Figure 1 shows the SIA system that was used to measure the saccharin contents. It consists of a Cavro syringe pump (Tecan Group Ltd., Männedorf, Switzerland), a series port electrical selection valve (Knauer GmbH, Köln, Germany), and a fiber optic spectrophotometer (Model S2000; Ocean Optics Inc., Largo, FL, USA) connected to a flow-through quartz cell with an inner volume of 450 µL and an optical path length of 10 mm (Hellma GmbH, Müllheim, Germany), and a deuterium-tungsten lamp (DH-2000, Ocean Optics Inc., Westfield, MA, USA) with two 2-m-long optical fibers with a fiber diameter of 200 µm. A double syringe pump was employed to increase the systemic speed-up capacity. Pump 1 was equipped with a 5-mL syringe that drew up the carrier buffer solution and pushed the reagent-sample mixture within the holding coil to the reaction coil. The holding and reaction coils were twisted from a PEEK tube (1 mm i.d. and 1.6 mm o.d.) for a good dispersion of the reagents and the sample. The absorbance of the violet-red compound solution was detected at 420 nm. The control of the system was accomplished using a personal computer (PC) equipped with the PCI-6024E analog-to digital (A/D) interface board (National Instruments Co., Seoul, Korea). The software used for the data acquisition and the system control was written using the graphical programming language, LabVIEW^TM^ (vers. 6.1, National Instruments Co., Seoul, Korea). The operating sequence of the SIA system is described in Table 1. The cycle starts with the drawing up of the carrier buffer solution to the holding coil. Sequentially, the ethanol, hydrogen peroxide, and p-chloranil reagents were drawn up along with the sample into the holding coil. By the process of flow reversal, the stack zones were directed to the reaction coil and the detector. The passage of the colored product through the flow-through cell resulted in a transient signal that was recorded as the absorbance at 420 nm, which was proportional to the saccharin concentration.

### 2.4. Construction of the Calibration Curves

Aliquots of saccharin stock solution (comprising saccharin concentrations from 50–600 mg/L) were placed on the sample valve, parallel to the ethanol, 20 mM p-chloranil, and 0.50 mL of the H_2_O_2_ (7.10 M), as described in Figure 1. Then, the SIA system was run according to the operating sequence in Table 1, (i.e., the setup conditions are a buffer flow rate of 1.6 mL/min, a buffer volume of 2400 µL, and the absorbance that was measured at 420 nm). The calibration curves were prepared by plotting the absorbance against saccharin concentrations.

### 2.5. Reference Method

For the assessment of the saccharin contents, aliquots of each sample were analyzed via the HPLC method that is described by Weinert et al. [18]. The HPLC system used consists of the Jasco liquid chromatography (Jasco Co., Easton, MD, USA) equipped with the Jasco PU-980 pump, an UV-Vis detector set to 254 nm, and the Rheodyne 20-µL injector. The Phenomenex LC-18 analytical column with the parameters of 250 mm × 4.6 mm (i.d.) (Phenomenex Co., Torrance, CA, USA) was used with packing material of a 5-µm particle size. The mobile-phase consisted of a mixture of 20% (*v/v*) reagent-grade glacial acetic acid in water, which was buffered to a pH 3.0 using a saturated sodium acetate solution. Before the injection, the samples were filtered through the Millex-HV 0.45-µm membrane filter (Millipore Co., Temecula, CA, USA) and this was followed by an ultra-sonication. A calibration curve was also prepared using a standard concentration between 50 and 600 mg/L.

## 3. Results and Discussion

**Figure 2:**
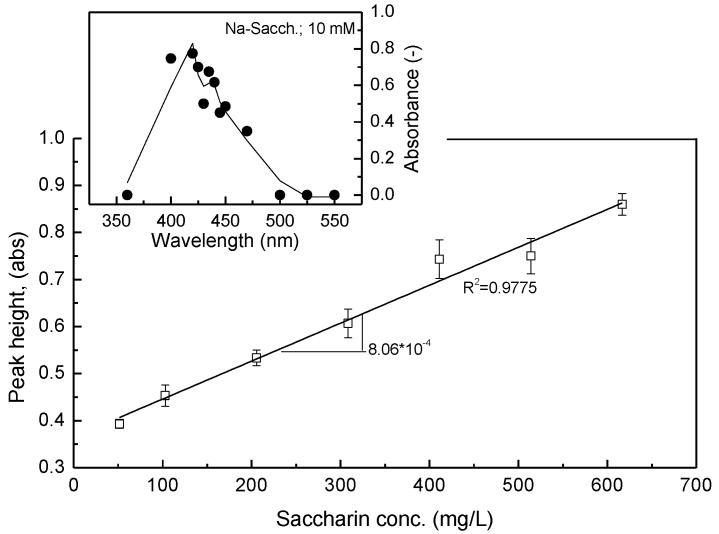
Linear calibration curve of saccharin under reference operating conditions (buffer flow rate and volume of 1.6 mL/min and 2400 µL, respectively; 975 µL of ethanol; 75 µL of H_2_O_2_ (7.10 M); 300 µL of p-chloranil (20 mM); 150 µL of saccharin).

The violet-red color development is based on the rapid formation of the complexes that is the result of the reaction of saccharin with p-chloranil (π acceptor) in the presence of H_2_O_2_ [18]. Gotardo et al. [19] also stated that the molecular interactions between the electron donors and the acceptors are generally associated with the formation of intensely colored charge-transfer complexes and radical ions, which absorb the radiation in the visible region of the spectrum (λ = 420 nm), as shown in Figure 2. The spontaneous formation of the violet-red color from the yellow appearance of the p-chloranil upon the reaction with the saccharin is sufficient evidence of the formation of a charge-transfer complex. This reaction is markedly accelerated by the presence of H_2_O_2_.

### 3.1. Characterization of the Saccharin-SIA System

The preliminary calibration curve of saccharin is presented in Figure 2. A considerably effective analytical curve (R^2^ = 0.9775) was obtained when the SIA system was run to measure the peak heights of seven standard saccharin solutions in the concentration range of 50.0–600.0 mg/L. The SIA system consists of the following reference conditional setup: (a) the buffer flow rate and volume are 1.6 mL/min and 2400 µL, respectively; and (b) the sequential order is 975 µL of ethanol, 75 µL of H_2_O_2_ (7.10 M), 300 µL of p-chloranil (20 mM), and then 150 µL of saccharin. Furthermore, to improve the correlation efficiency and achieve a zero point-crossing line by as much as possible, it was necessary to fix several of the treatments including the buffer flow rate and volume, the p-chloranil concentration, and the reactant volume (p-choranil and H_2_O_2_).

**Figure 3:**
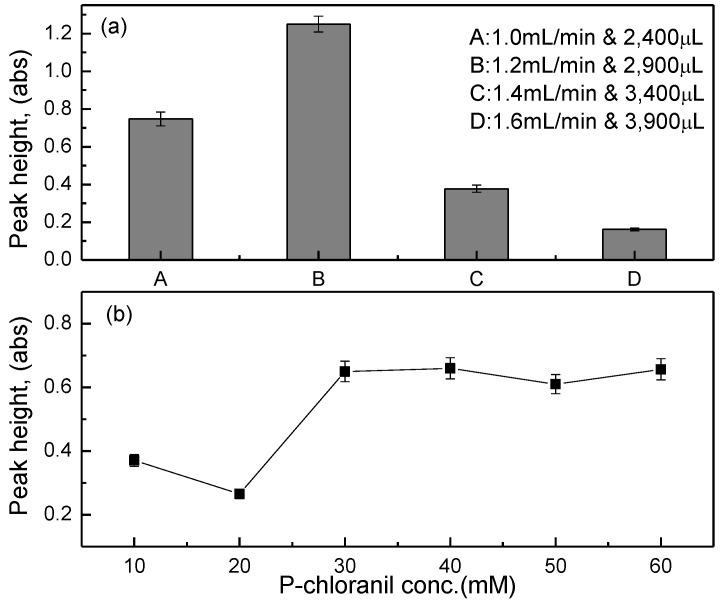
(**a**) Effects of the buffer flow rate and volume; and (**b**) effects of the p-chloranil concentration on the saccharin-SIA system under reference operating conditions with 10 mM sodium saccharin.

The volumetric flow rate is a key parameter that has a marked influence on the dispersion zone in a SIA manifold [16,20]. Figure 3a shows the effects of the buffer flow rate and volume on the peak height during the measurements of saccharin concentration. The highest peak height was achieved with the buffer flow rate of 1.2 mL/min and the buffer volume of 2900 µL, and the draw-up time of this setup is ~145 s. Weinert et al. [18] used a simplified spectrophotometric method for a routine analysis of the saccharin concentration in commercial non-caloric sweeteners, reporting that a complete color development was attained after 8 min, while the color stability lasted for 20 min.

The effects of the p-chloranil concentrations on the peak height during the saccharin measurements are described in Figure 3b. The optimal p-chloranil concentration was determined as 30 mM, even though the resultant peak heights are slightly lower than those at the concentrations of 50 and 60 mM. Based on several runs of the SIA system, a p-chloranil concentration that is more than 30 mM caused the formation of microparticles in the flow-through cell. This p-chloranil concentration is admittedly very high, compared with 4 mM that was reported by Weinart et al. [18]; however, it is considered as reasonable for a sufficiently short reaction time. Ruzicka and Hansen [21] also ruled that a good dispersion zone could be achieved with either the use of a high reagent concentration or an increasing of the injected sample volume.

**Figure 4:**
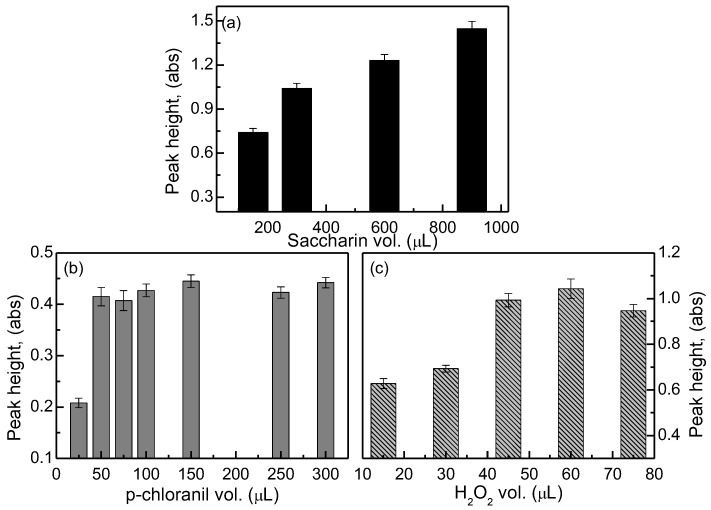
Effects of the reactant volumes on a saccharin-SIA system under reference operating conditions: (**a**) saccharin volume; (**b**) p-chloranil volume; and (**c**) hydrogen peroxide (H_2_O_2_) volumes.

The effects of the reactant volumes on the peak height during the measurements of saccharin concentrations are described in Figure 4. The optimal sample (saccharin) volume for the yielding of a high peak height is 900 µL, as shown in Figure 4a. In comparison with the p-chloranil volume of 300 µL, it could be assumed that the appropriate volume ratio of the sample (saccharin) to p-chloranil is 3 to 1. Ruzicka and Hansen [21] stated that a powerful way to change the dispersion to increase the peak height and sensitivity is the increasing of the volume of the injected sample solution.

Regarding the effects of the p-chloranil volume that are shown in Figure 4b, the optimal value was determined as 150 µL, even though its peak height did not differ from the peak heights of the volumes ranging from 50–300 µL. The main consideration for this decision is the smallest relative standard deviation (RSD) that was produced by the p-chloranil volume of 150 µL. The volume of 150 µL was also selected because its correlation seemed to be positive based on the previous tests on the effects of the p-chloranil concentration on the peak height; that is, this volume would be influential on the consumption of the p-chloranil volume. Another consequence is the subsequent use of a saccharin volume of 450 µL that is in consideration of the optimal volume ratio of 3 to 1. Figure 4c shows that significantly high peak heights were obtained with the H_2_O_2_ volumes of 15, 30, and 45 µL. With a similar consideration of the smallest RSD, the H_2_O_2_ volume of 30 µL was deemed as the optimal level. Moreover, for volumes that are less than 30 µL, the sequential injection-graphs are approximate (data not shown).

**Figure 5:**
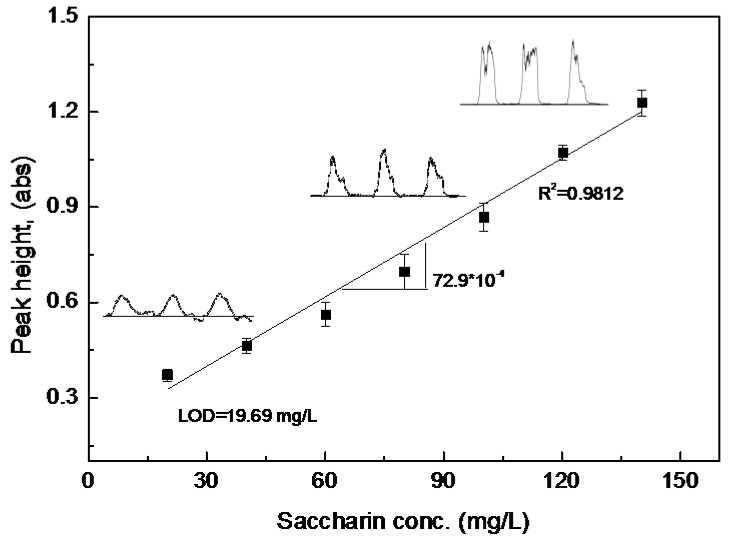
Calibration curve of a saccharin-SIA system under optimized operating conditions (buffer flow rate and volume of 1.2 mL/min and 2900 µL, respectively; 870 µL of ethanol; 30 µL of H_2_O_2_ (7.10 M); 150 µL of p-chloranil (30 mM) and 450 µL of saccharin).

The developed analytical method was then validated by the construction of a calibration curve, whereby a limit of detection (LOD) was defined. Under the previously stated optimized operating conditions, the regression line was obeyed in the concentration range of 20–140 mg/L with a good correlation coefficient (R^2^ = 0.9812), as can be seen in Figure 5. The LOD (3·(Sy/x)/slope of calibration curve) is 19.69 mg/L. The SIA method also showed high reproducibility with small values of RSD, that is, 6.4% at 40 mg/L, 7.3% at 100 mg/L and 6.8% at 140 mg/L. The produced concentration range showed a considerable consistency with those presented in other studies of the flow injection method for the determination of saccharin concentration (i.e., 10–100 mg/L [10], 1.0–200.0 μg/mL [15], and 5–75 μg/mL [14]).

### 3.2. Analytical Application

The proposed SIA method was applied to determine the saccharin concentrations in different food products containing saccharin. The samples were analyzed using both the proposed SIA method and the HPLC method [18], and their results were then compared (Table 2). The average recovery percentages of the proposed SIA method are in the range from 93.1–111.5%. Capitán-Vallvey et al. [15] yielded saccharin recovery percentages ranging from 100.3–105.3%. Meanwhile, Kritsunankula et al. [10] and Yebra et al. [14] reported acceptable saccharin recovery percentages in several commercial liquid foods and sweetener mixtures, respectively, after the completion of flow injection determination processes.

## 4. Conclusions

A simple, rapid, and cost-effective sequential injection analysis (SIA) method was successfully optimized for the determination of saccharin (C_7_H_5_NO_3_S) in food products. The optimal setup of this system occurred with a buffer flow rate of 1.2 mL/min and a buffer volume of 2900 µL. The draw-up time of this setup is ~145 s. After an investigation of the effects of the p-chloranil concentration and the reactant volumes, the optimally ordered sequence of the reagent concentration and volumes is 870 µL of ethanol, 30 µL of H_2_O_2_ (7.10 M), 150 µL of p-chloranil (30 mM), and 450 µL of saccharin. This SIA configuration produced a good linear calibration curve with a correlation coefficient (R^2^ = 0.9812) in the saccharin concentration range of 20–140 mg/L and with a detection limit of 19.69 mg/L. The analytical applications in the different food categories also showed acceptable saccharin recovery percentages in the range of 93.1–111.5%.

## Figures and Tables

**Table 1:** Operating sequence of the SIA system for the determination of saccharin concentration.

Time (s)	Pump 1	Pump 2	Valve	Description
0	Reverse	Off		Drawn up carrier buffer solution
30	Off		Position 2	Pump-stop, select solvent
35		Reverse	Position 2	Drawn up solvent (ethanol)
68		Off	Position 1	Pump-stop, select assay line
				(to HC = holding coil)
73		Reverse	Position 3	Drawn up reagent 1 (H_2_O_2_)
89		Off	Position 1	Pump-stop, select assay line
				(to HC = holding coil)
94		Reverse	Position 4	Drawn-up reagent 2 (p-chloranil)
116		Off	Position 1	Pump-stop, select assay line
				(to HC = holding coil)
121		Reverse	Position 5	Drawn-up sample solution (saccharin)
132		Off	Position 1	Pump-stop
137	Forward		Position 1	Pump stack of zones to the reactor
				(RC = reaction coil) and detector
282	Off			Pump-stop
287		Forward	Position 1	Pump stack of zones to the reactor
				(RC = reaction coil) and detector
332		Off		Pump-stop
337			Position 2	Return to starting position

**Table 2:** Determination of sodium saccharin in artificial sweeteners using the proposed SIA method and the high-performance liquid chromatography (HPLC) method.

Sample	Labeled ^a^	Proposed SIA Method		HPLC Method	
		Found ^d^	Recovery (%) ^d^	Found ^d^	Recovery (%) ^d^
Food sweetener ^b^	50,000.0	55,774.0 ± 2946.0	111.5 ± 5.9	46,170.7 ± 616.0	92.3 ± 1.2
Food in brine ^b^	500.0	496.1 ± 33.7	99.2 ± 6.7	554.3 ± 79.0	110.9 ± 15.8
Soft drink 500 ^c^	500.0	465.4 ± 14.1	93.1 ± 2.8	550.6 ± 7.6	110.1 ± 1.5
Pudding 500 ^c^	500.0	477.9 ± 34.2	95.6 ± 6.8	528.3 ± 25.7	105.7 ± 5.1
Steamed-bread 500 ^c^	500.0	535.0 ± 74.0	107.0 ± 14.8	517.56 ± 36.0	103.5 ± 7.2

^a^ Amount of sodium saccharin measured in mg/kg for the solid samples and measured in mg/L for the liquid samples. ^b^ Commercial food collected from local market. ^c^ Home-made samples spiked with 500 mg/kg of saccharin (Appendix). ^d^ Average ± standard deviation (SD) of three determinations per sample.
